# Design, synthesis and applications of a coumarin based fluorescent probe for selective detection of hypochlorite ions and live cell imaging

**DOI:** 10.1039/d5ra06003f

**Published:** 2025-10-30

**Authors:** Wajeeha Zareen, Nadeem Ahmed, Mostafa A. Ismail, Hafza Feroz, Muhammad Ali Khan, Zahid Shafiq, Sobhi M. Gomha

**Affiliations:** a Institute of Chemical Sciences, Bahauddin Zakariya University Multan 60800 Pakistan zahidshafiq@bzu.edu.pk; b College of Chemistry & Chemical Engineering, Central South University Changsha Hunan 410083 China; c Department of Chemistry, Faculty of Science, Research Center for Advanced Materials Science (RCAMS), King Khalid University P.O. Box 960 Abha 61421 Saudi Arabia; d Department of Chemistry, Faculty of Science, Islamic University of Madinah Madinah 42351 Saudi Arabia smgomha@iu.edu.sa

## Abstract

The immune system's protection against invasive microorganisms and other biological functions in living things depend heavily on hypochlorous acid (HOCl). For the sensitive detection of HOCl, a turn-on fluorescent probe (W-HOCl) has been developed. The probe is well characterized by ^1^HNMR, ^13^CNMR, FTIR and HRMS. Through the oxidation of sulfide to sulfoxide caused by HOCl, the probe quickly identifies HOCl. The fluorescence was enhanced 42 times as a result of the reaction between W-HOCl and HOCl. The probe has excellent photophysical properties along with a detection limit as low as 6 nM. The imaging of HOCl in living cells showed the probe's potential as an HOCl biosensor. Therefore, the probe is predicted to be useful for the accurate detection of HOCl in complex biosystems.

## Abstract 2

The immune system's protection against invasive microorganisms and other biological functions in living things depend heavily on hypochlorous acid (HOCl).

## Graphical abstract

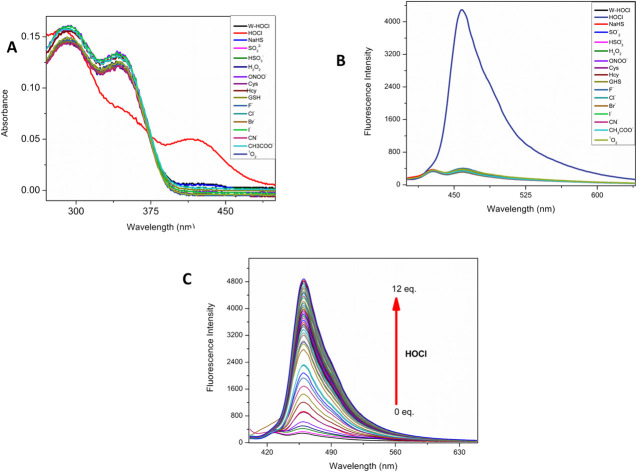

## 1. Introduction

In recent years, there has been growing interest in the function of anions in biology, industry, medicine, and the environment. Anion sensors with remarkable design and synthesis capabilities have attracted the attention of experts in inorganic catalysis, pharmaceutical synthesis, environmental analysis, and life sciences. Many fluorescent sensors have been reported for visualizing viscosity in dysfunction, inflammation, and Alzheimer's disease, and an allosteric hairpin switch-based isothermal amplification strategy for sensitive detection of bacterial detection.^1–3^ Hypochlorous acid (HOCl)/hypochlorite (ClO^−^) is a very active oxidation species that is essential for both environmental safety and biomedicine.^4,5^ As is well known, we utilize HOCl to disinfect and purify water in our daily lives by getting rid of bacteria. Myeloperoxidase (MPO) catalyzes the reaction of hydrogen peroxide with chloride ions to form HOCl in biological systems.^6^ It is essential for preserving the intracellular redox equilibrium, reducing inflammation, and fending off infections.^7,8^ However, too much HOCl can result in the production of dangerous substances in water (such as trihalomethanes) and cause a number of oxidative stress-related illnesses in people, such as cancer, arthritis, cardiovascular disease, and neurodegeneration. In living things, highly active HOCl can aid in the chemical alteration and destruction of a variety of biomolecules, such as proteins, lipids, and nucleic acids.^9–14^ Thus, it is necessary to develop an efficient technique for the real-time detection of HOCl/ClO in biological and environmental systems.

Small-molecule fluorescent probes are highly sensitive, noninvasive, and detect in real time, they have attracted a lot of interest.^15–26^ Using *p*-methoxyphenol as the recognition group, Yang's group discovered in 2008 that hypochlorous acid could be specifically detected in a variety of ROS.^27^ A number of fluorescent probes for the detection of hypochlorous acid were thereafter published. These probes were made in accordance with several response mechanisms, including the oxidation of *p*-methoxyphenol described above, oxidative removal of oximes, cyclization of thioesters and amides following chlorination, double bond oxidation, oxidation of chalcogenides (S, Se, Te), and other processes.^28–39^ Low sensitivity and a lengthy response time are two drawbacks of the available HOCl fluorescent probes, which also have rather simple functionalities.

Herein we have synthesized a coumarin based probe W-HOCl. The probe works on oxidation of sulfide to sulfoxide. We have added thiomorpholine moiety to coumarin fluorophore as a result the spectral properties of coumarin is quenched due to PET process. When probe is treated with HOCl the PET process is inhibited as a result spectral property of coumarin derivative is restored. Overall, the probe's excellent sensitivity and selectivity, as well as its quick fluorescence response to HOCl, make it suitable for use as a tool for detecting HOCl in living organisms.

## 2. Experimental section

### 2.1. Material

All chemicals such as Meldrum's acid, 2-hydroxy-4-methoxybenzaldehyde, piperidine, thiomorpholine, absolute ethanol, dichloromethane, methanol, 1-ethyl-3-(3-dimethylaminopropyl) carbodiimide [EDCI], and dimethyl amino pyridine [DAMP] were purchased from Sigma-Aldrich company and directly used without further purification. MCF-7 cells were obtained from Xiangya Hospital at Central South University, China.^40^ Other chemical including, Cys, Hcy, GSH, H_2_O_2_, NaOCl, NaHS, NaHSO_3_, ONOO^−^, Na_2_SO_3_, NaF, NaI, KBr, NaCl, CH_3_COOH, HCN, were purchased from admass company for the preparation of analytes for spectral studies.

### 2.2. Cell imaging

MCF-7 cells were grown at 37 °C in an incubator with 5% CO_2_ in Dulbecco's Modified Eagle Medium (DMEM) supplemented with 10% fetal bovine serum. Probe W-HOCl (10 μM) was introduced to MCF-7 cells for imaging, and the cells were then incubated for 30 minutes at 37 °C in an incubator with 5% CO_2_. They were cleaned three times using PBS buffer (pH 7.4) solution, then treated once more with HOCl solution and incubated for one hour in an incubator with 5% CO_2_. PBS buffer (pH 7.4) was used three times to wash the cells. The Leica TCS SP8 confocal microscope was then used to image the cells.

### 2.3. Synthesis of probe

**Scheme 1:**
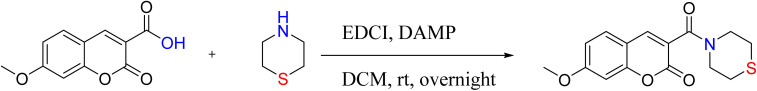
Synthesis of probe W-HOCl.

Coumarin is synthesized by Knoevenagel condensation reaction of 2-hydroxy-4-methoxybenzaldehyde and Meldrum's acid in presence of base (piperidine) in absolute ethanol (Scheme 1). The reaction mixture is refluxed overnight and After completion of the reaction, confirmed by TLC, the mixture was cooled, and the formed precipitate was collected by filtration which were used in the next step without further purification. The probe W-HOCl is synthesized by the reaction of 7-methoxy-2-oxo-2*H*-chromene-3-carboxylic acid (0.3 g, 1.36 mmol) thiomorpholine (0.28 g, 2.72 mmol), EDCI (0.27 g, 1.76 mmol) and DMAP (10 mg, 0.45 mmol) in dry DCM. The reaction mixture is stirred at rt overnight. The progress of reaction is checked by TLC and after confirmation of reaction the reaction is quenched with water and extracted with DCM. The organic layer is combined and dried over Na_2_SO_4_. The solvent is removed by rotary evaporator and crude product is purified by column using DCM : MeOH (20 : 1). The off-white solid is obtained in a good yield of 82%. Melting point 180–182 °C. IR O–C=O and N=CO, 1750–1600; C–O–C, 1250–1050; C–S–C 1300–1100. ^1^H NMR (400 MHz, CDCl_3_) *δ* 7.86 (s, 1H), 7.43 (d, *J* = 8.6 Hz, 1H), 6.92–6.80 (m, 2H), 4.08–3.97 (m, 2H), 3.90 (s, 3H), 3.65 (t, *J* = 5.1 Hz, 2H), 2.73 (q, *J* = 6.7 Hz, 4H). ^13^C NMR (101 MHz, CDCl_3_) *δ* 164.3, 163.9, 158.3, 156.2, 143.6, 129.6, 121.2, 113.3, 111.9, 100.7, 77.4, 77.3, 77.1, 76.8, 55.9, 50.0, 44.7, 27.8, 27.4. Calculated mass for C_15_H_15_NNaO_4_S^+^ [M + Na]^+^, 328.0614. Found, 328.0688.

## 3. Result and discussion

### 3.1. W-HOCl characterization

The probe W-HOCl is synthesized by coupling of thiomorpholine and 7-methoxy-2-oxo-2*H*-chromene-3-carboxylic acid in a good yield. The probe is characterized by ^1^HNMR, ^13^CNMR, IR and HRMS (SI 1–4). The thiomorpholine is a responsive site for HOCl. The thioether is converted to sulfoxide as a result the fluorescence of probe is enhanced as reported earlier.^41–45^ The stability of probe is checked through the ^1^HNMR as well as cycling test SI-9. In cycling test, the probe is excited at 400 nm. There is a negligible change in intensity this indicate that probe is high stable. Similarly, after the addition of HOCl probe showed an enhancement in intensity and then probe is excited at same excitation wavelength again there is no change in intensity. Which indicate excellent stability of probe after the addition of HOCl. This probe could be used to detect HOCl where there are chances of degradation of probes.

### 3.2. Response of W-HOCl toward HOCl

**Fig. 1:**
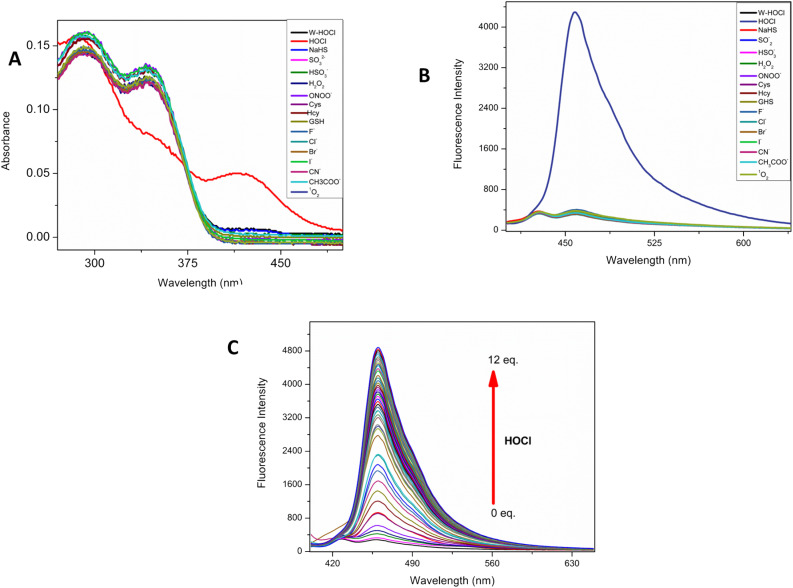
(A) The electronic absorption spectra of probe W-HOCl (10 μM) (B) *F*_l_ selectivity of probe (C) *F*_l_ titration of probe with HOCl (10 eq.) in HEPES buffer (10 mM, pH = 7.4, having 60% EtOH).

After treating probe W-HOCl (10 μM) in 10 mM HEPES buffer having 60% EtOH with HOCl the electronic spectra were acquired after 5 minutes of reactions in order to examine the probe's UV-vis absorption and fluorescence responses to HOCl. The probe without the addition of HOCl had two absorbance band one at 346 nm and other one was at 290 nm as shown in Fig. 1A. When probe was treated with HOCl, the absorbance band at 346 nm was decreased and a new band at 422 nm was emerged. Also, the other band showed a decreased in wavelength and shifted 285 nm. There is a slight color change after the addition of HOCl to the probe solution SI-7. In pure DMSO-d_6_ the color is more changed because the quantity of sample is more (20 mg) as well as this one is pure DMSO-d_6_ alone. In the other picture the sample is prepared as for spectral studies which also indicate a slight change in color that can be views by naked eyes.

When the probe is excited at 380 nm it has a weak fluorescence at 460 nm (Stokes shift 80 nm), when probe was treated with HOCl as shown in Fig. 1B, the fluorescence intensity of probe was emerged at 460 nm. There was no change in fluorescence intensity with other interfering anions. This result indicates high selectivity of probe toward HOCl. The interaction of probe with HOCl is further confirmed by titration method as shown in Fig. 1C the fluorescence of probe was consistently increased with gradual addition of HOCl solution to probe solution. There was a good linear relationship in addition of HOCl and probe response (SI-5). After the addition of 12 eq. of HOCl the saturation point of the probe was reached, at this point the further addition of HOCl show no change in emission intensity. The detection limit of W-HOCl for HOCl was then found to be 6 nM using the IUPAC technique (3*σ*/*k*). The probe has some advantages over the other reported probes Table 2.

### 3.3. Competitive study

**Fig. 2:**
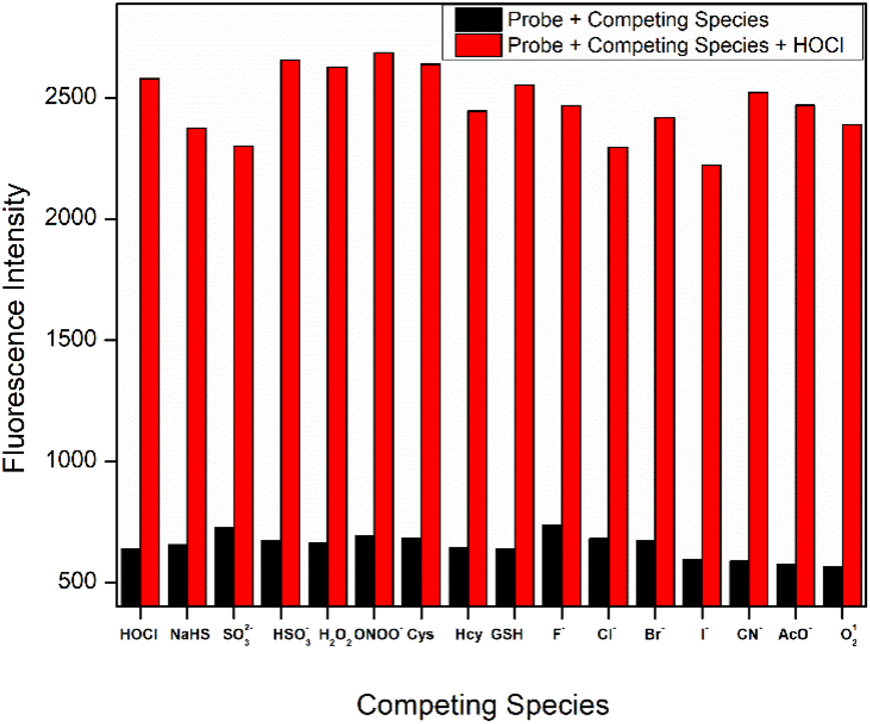
FL response of W-HOCl (10 μM) with HOCl (10 eq.) at 460 nm changes upon addition of ions in the presence of HOCl (10 eq.) in HEPES buffer (10 mM, pH = 7.4, having 60% EtOH, *λ*_ex_ = 380 nm, slit: 5 nm).

For the probe to potentially be used in complicated systems, its strong selectivity for HOCl was crucial. Thus, we examined the change in fluorescence intensity of probe following the addition of HOCl (50 μM) and other interfering species. After other interfering analytes were added, Fig. 2 demonstrates that the probe's fluorescence intensity hardly changed. After adding HOCl, however, the probe's fluorescence intensity showed a very noticeable change. The findings of the aforementioned experiment showed that the probe had a strong selectivity for HOCl. The selective sensing of probe is further verified by conducting fluorescence of probe in presence of HOCl and Na_2_SO_3_. We know that Na_2_SO_3_ can react with HOCl to form HCl and Na_2_SO_4_. So, we have prepared two sample in one sample we have added HOCl and in another sample we have added HOCl and Na_2_SO_3_ together. After 20 mints the probe solution is added to them and fluorescence is conducted after 10 mints, there is clear decrease in fluorescence intensity of the sample having HOCl and Na_2_SO_3_. This result indicates that the enhancement in fluorescence intensity is due to the response of HOCl SI-10.

### 3.4. Time and pH response of probe

**Fig. 3:**
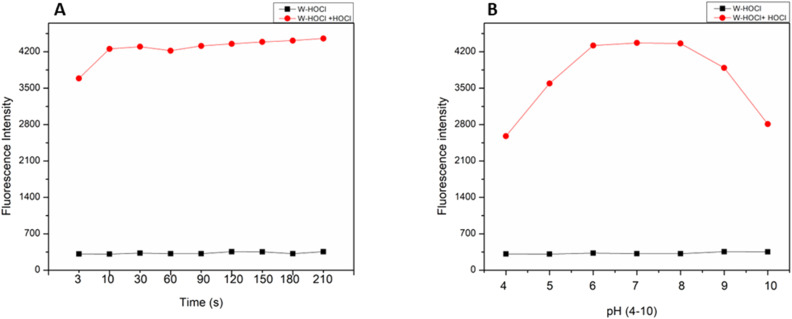
FL response of W-HOCl (10 μM) at 460 nm when treated with HOCl ions (10 eq.) (A) with time (B) at different pH in HEPES buffer (10 mM, pH = 7.4, having 60% EtOH, *λ*_ex_ = 380 nm, slit: 5 nm).

By monitoring the fluorescence intensity of the probe at 460 nm after injecting HOCl (100 μM) over time, the time-dependent fluorescence response of the probe to HOCl was investigated (Fig. 3A). At 460 nm, the probe's fluorescence intensity dramatically increased after 100 μM HOCl was added, and it plateaued at 10 s. The aforementioned experimental findings demonstrate that the probe had good stability prior to the addition of HOCl and was able to determine HOCl in 10 s, which was a comparatively quick reaction time when compared to those previously documented in the literature. This study examined how the fluorescence intensity of the probe (10 μM) changed in various pH settings before and after the addition of HOCl (100 μM) (Fig. 3B). Fig. 3B shows that the fluorescence intensity of the probe did not significantly change prior to the addition of HOCl, indicating that the probe was stable in the pH 4.00–10.00 range. However, the fluorescence intensity was greatly increased in the pH 4.00–10.00 range following the addition of HOCl, indicating that the probe could detect HOCl under physiological conditions.

### 3.5. Real water sample

It is very important to detect hypochlorous acid in the water environment since high levels of HOCl can pollute the water and kill aquatic life. In light of the aforementioned considerations and the spectral test results, we were inspired to use W-HOCl for the detection of HOCl in real-world water samples (Table 1). In this work, two water samples were obtained, and after the large impurities were removed, the treated water sample was subjected to W-HOCl analysis. According to the findings of the study on HOCl-spiked drinking and river water, W-HOCl can be used as a tool for HOCl detection in water with high precision and accuracy. All detections had recoveries between 99.17 and 102.30 percent and a relative standard deviation (RSD) of less than 2.99%.

**Table 1:** Using W-HOCl (10 μM) as a probe, HOCl can be found in water samples

Sample	HOCl added	HOCl found	RSD (% *n* = 3)	Recovery (%)
Tap water	30	35.3	1.93	101.59
	50	55.59	2.23	101.46
	70	75.07	1.09	99.10
River water	30	30.59	2.16	102.23
	50	49.59	2.19	99.18
	70	70.13	2.99	100.62

**Table 2:** Comparison of probe W-HOCl with other reported work

Probe	Structure	Detection limit (nM)	Daily life application	Reference
1	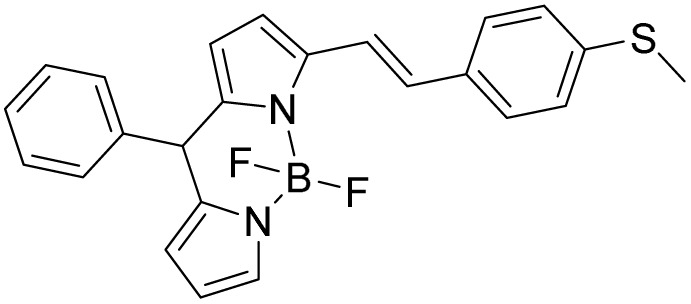	30	No real sample	46
2	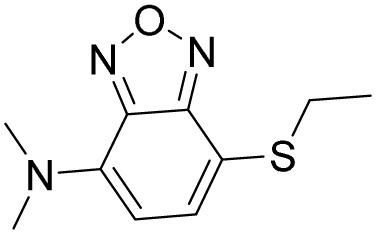	34	No real sample	47
3	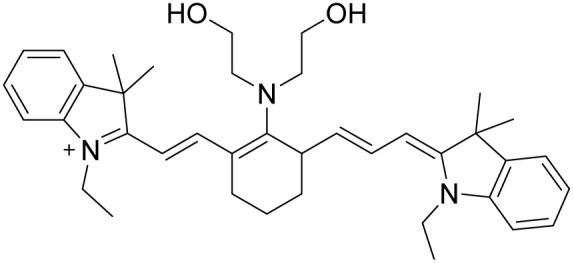	22	No real sample	48
4	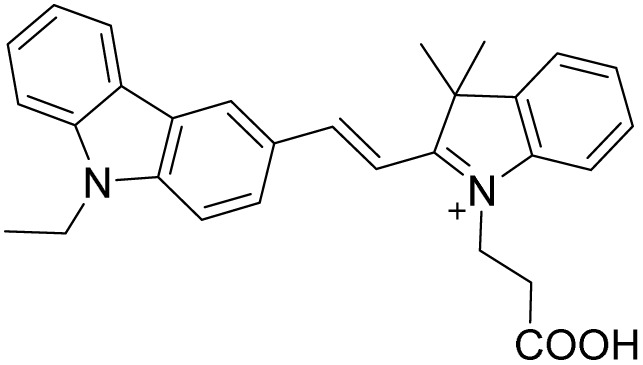	444.7	No real sample	49
5	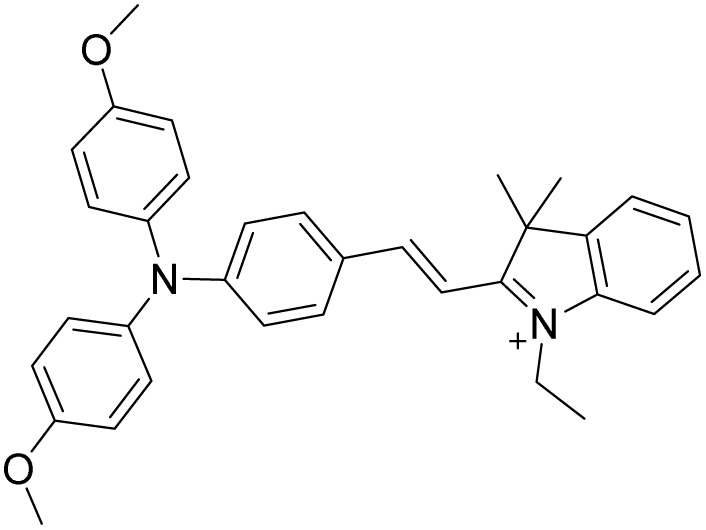	13.2	No real sample	50
6	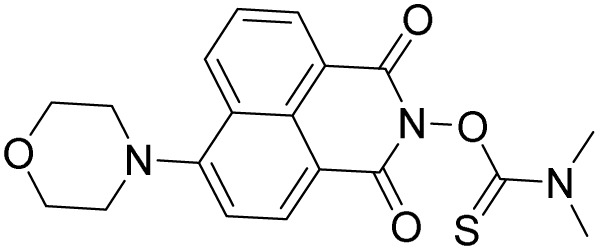	105.24	No real sample	51
This work	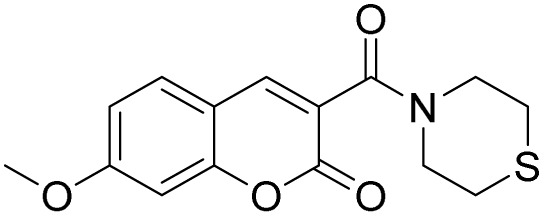	6	Real sample	

### 3.6. Sensing mechanism

**Fig. 4:**
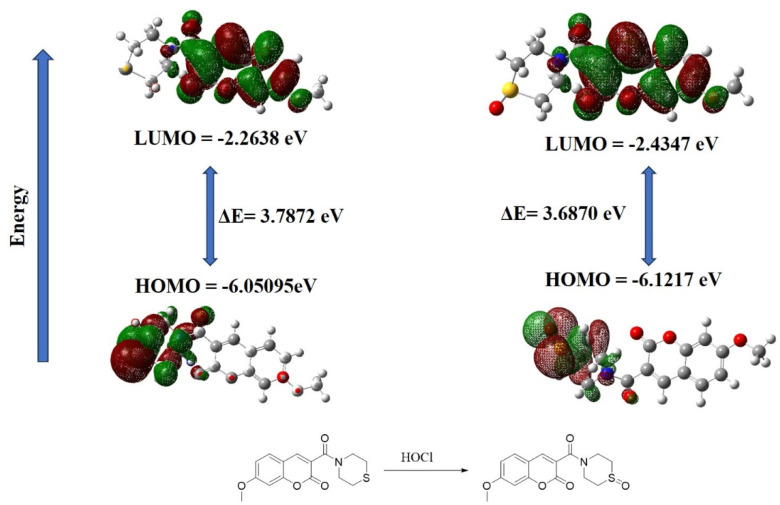
HOMO–LUMO energy gap of probe W-HOCl before and after addition of HOCl.

Density functional theory calculations were used to further clarify the fluorescence response mechanism of W-HOCl [Fig. 4]. The observed redshift in the UV-vis absorption spectra of probe W-HOCl upon the addition of HOCl to generate W-HOCl-SO is consistent with the HOMO–LUMO energy gap of W-HOCl-SO (Δ*E* = 3.8670 eV) being smaller than that of the probe itself (Δ*E* = 3.7872 eV). But sulfur's (S) heavy atom action caused the fluorescence of W-HOCl to be extinguished. The oxidation of sulfur caused by the HOCl treatment of the probe produced W-HOCl-SO, which showed a notable emission of fluorescence. This behavior is explained by the d-PET effect, or donor-photoinduced electron transfer.

**Scheme 2:**
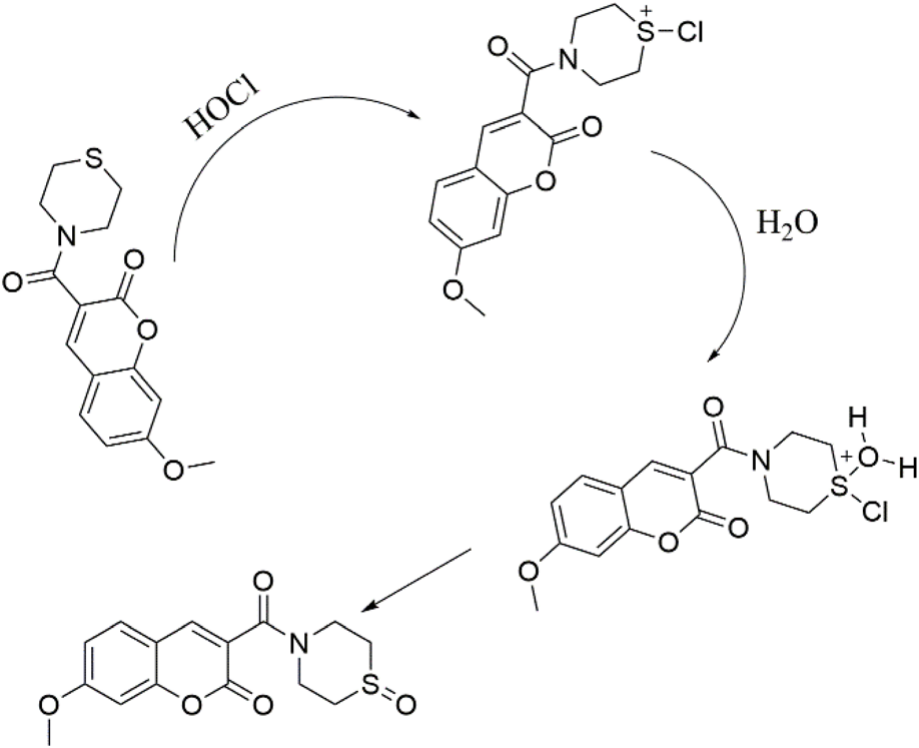
Proposed sensing mechanism.

Additionally, the existence of W-HOCl-SO in the reaction solution was verified by high-resolution mass spectrometry (SI-6). The probe itself has mass 328.0688 (C_15_H_15_NNaO_4_S^+^), after the addition of HOCl a clear peak of mass 344.3242 (C_15_H_15_NNaO_5_S^+^) is found which is due to the oxidation of Sulfur to sulfoxide. In light of these results, we suggest the response mechanism shown in Scheme 2. The ^1^HNMR result of probe after the addition of HOCl confirm the mechanism is through oxidation of sulfide to sulfoxide, rather than the breakdown of ring or chlorination SI-8.

### 3.7. Cytotoxicity testing

**Fig. 5:**
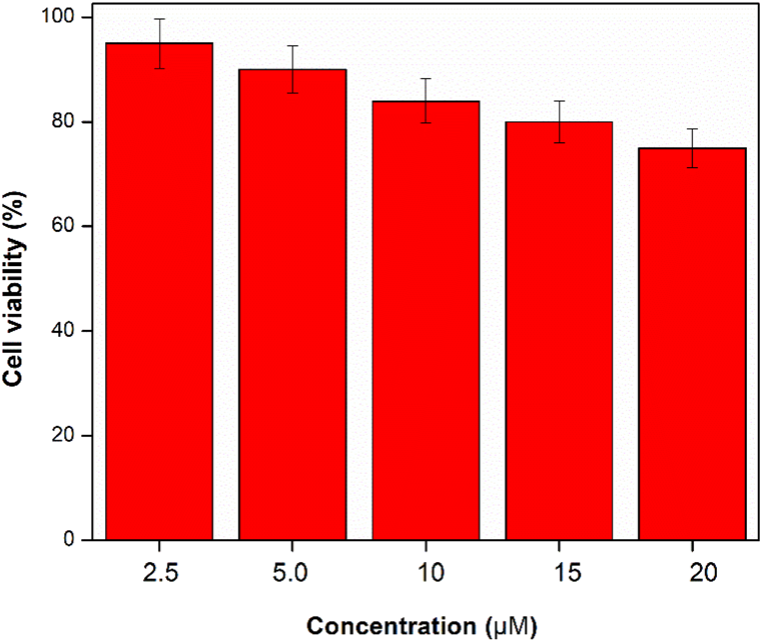
Toxicity assay of probe W-HOCl at different concentration in MCF-7 cells.

**Fig. 6:**
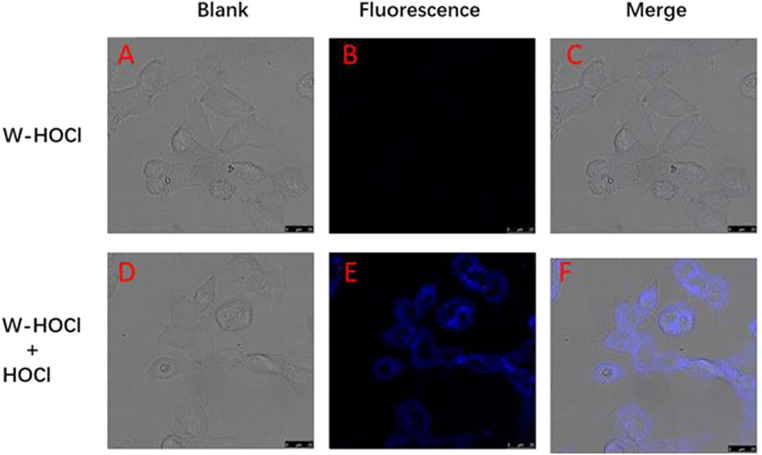
Confocal fluorescence images of HOCl with probe W-HOCl in MCF-7 cells. (A and B) MCF-7 cells pretreated with W-HOCl (10 μM) for 30 min. (D and E) MCF-7 cells were pretreated with probe W-HOCl (10 μM) for 30 min and then incubated with HOCl (10 equiv.) for 1 h. (C) Combined image of (A) with (B). (F) Combined image of (D) with (E) (excitation: 380 nm, emission: 495 nm).

The MTT method was used to evaluate the probe's cell toxicity on MCF-7 cells in order to determine whether it could be used in organisms (Fig. 5). Fig. 6 shows that after the MCF-7 cells were incubated for 24 hours with different concentrations of the probe (0, 2, 4, 8, 16 μM), the cell viability exceeded 80%. The aforementioned experimental data showed that the probe exhibited nearly minimal cytotoxicity towards MCF-7 cells.

### 3.8. Confocal imaging in living cells

Subsequently, HOCl in living MCF-7 cells were then traced using probe W-HOCl (Fig. 6). The MCF-7 cells were treated for 30 minutes at 37 °C in 5% CO_2_ with 10 μM probe W-HOCl. There was no fluorescence in blue channel (Fig. 6A–C). When 100 μM HOCl was added and incubated for an additional hour, a clear and intense fluorescence was seen, as predicted (Fig. 6D–F). This outcome demonstrates the probe's value in identifying HOCl in live cells.

**Fig. 7:**
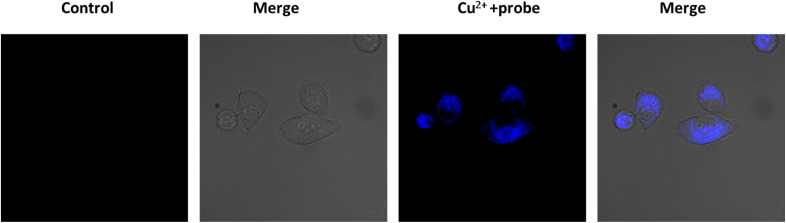
Generation of ROS in cell through copper promoted death.

Excess level of copper ions causes cell death and as a result ROS are produced. By using this probe W-HOCl we have confirmed this phenomenon. Firstly, we have added copper to the cell there is no fluorescence in blue channel but after incubating with probe there is fluorescence in blue channel which showed that after excess level of copper there is production of ROS. This probe is useful to detect the dead cell Fig. 7.

## 4. Conclusion

In summary, a fluorescence probe W-HOCl is developed for selective and efficient detection of HOCl. The probe was designed by functionalization of a coumarin by thiomorpholine group, and demonstrated turn-on fluorescence while responding to HOCl. The probe has excellent photophysical properties along with detection limit as low as 6 nM. The recoveries of HOCl were in the range of 99.17–102.30% followed by relative standard deviation (RSD) detections less than 2.99%. The successful imaging of HOCl in live cells and water sample detection further demonstrated the practical applications of the probe in biological systems.

## Author contributions

Wajeeha Zareen, Hafza Feroz: investigation, formal analysis. Nadeem Ahmed: writing – original draft, validation, data curation. Mostafa A. Ismail: formal analysis, software, data curation. Zahid Shafiq: writing – original draft, supervision, conceptualization. Muhammad Ali Khan: supervision, conceptualization. Sobhi M. Gomha: formal analysis, data curation, funding acquisition.

## Conflicts of interest

The authors declare that they have no known competing financial interests or personal relationships that could have appeared to influence the work reported in this paper.

## Supplementary Material

RA-015-D5RA06003F-s001

## Data Availability

The data supporting this article have been included as part of the supplementary information (SI). Supplementary information is available. See DOI: https://doi.org/10.1039/d5ra06003f.
