# Supplementary material for: Design, synthesis and applications of a coumarin based fluorescent probe for selective detection of hypochlorite ions and live cell imaging
Source: RSC Adv. 2025 Oct 30;15(49):41691–8. doi: 10.1039/d5ra06003f (PMC12573239; doi:10.1039/d5ra06003f)
Supplement: RA-015-D5RA06003F-s001 [file RA-015-D5RA06003F-s001.pdf]

Supplementary Information (SI) for RSC Advances.
This journal is © The Royal Society of Chemistry 2025

Table of content

1. ^{1}HNMR of probe W-HOCl……………………………………………….…………SI-1
2. ^{13}CNMR of probe W-HOCl………………….…………………………………...SI-2
3. IR of the probe W-HOCl………………………………………………………..…..SI-3
4. HRMS of probe W-HOCl…………………………………………………..……..SI-4
5. Linear plot of probe W-HOCl vs HOCl…………………………………….…...….SI-5
6. HRMS of probe W-HOCl after addition of HOCl………………………………..….SI-6
7. Change in color after the addition of HOCl to probe solution and HNMR sample…..SI-7
8. Stability and proposed mechanism confirmation of probe through HNMR…………...SI-8
9. Cycling test…………………………...…………………………………..………SI-9

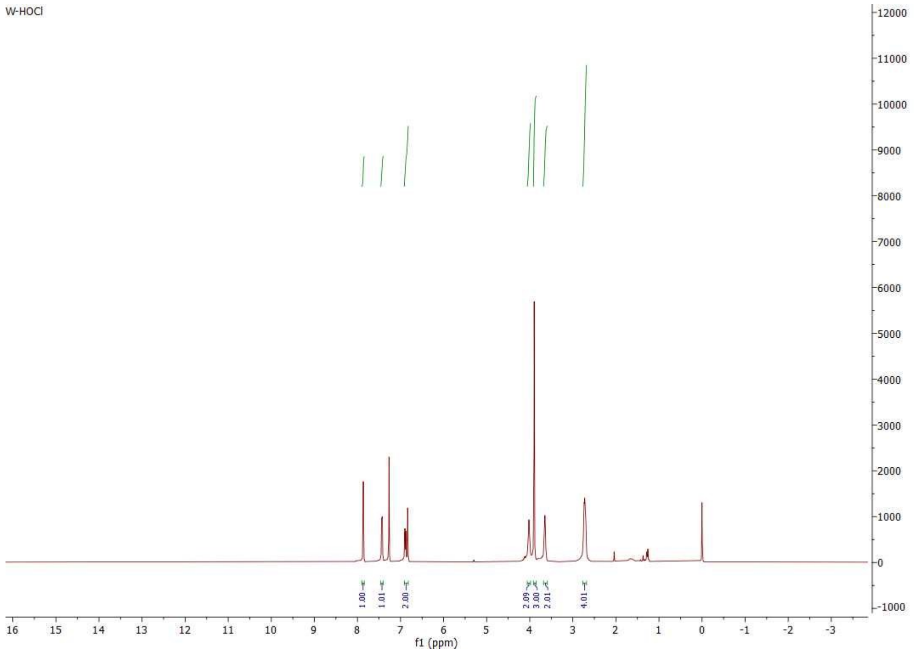

SI-1- ^{1}HNMR of W-HOCl in $CDCl_3$

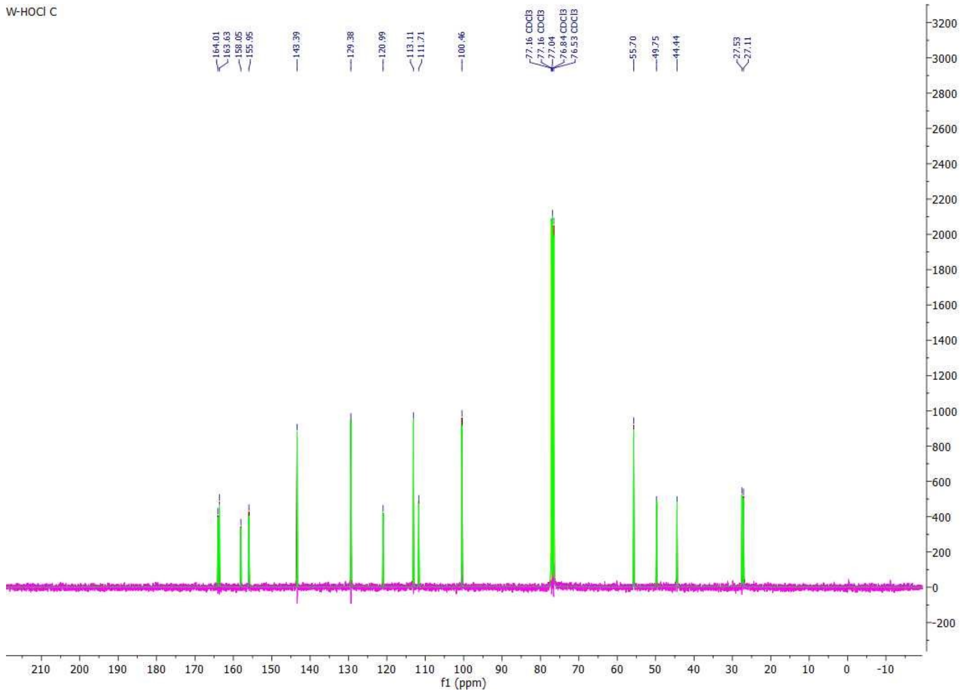

SI-2- ^{13}CNMR of W-HOCl in $CDCl_3$

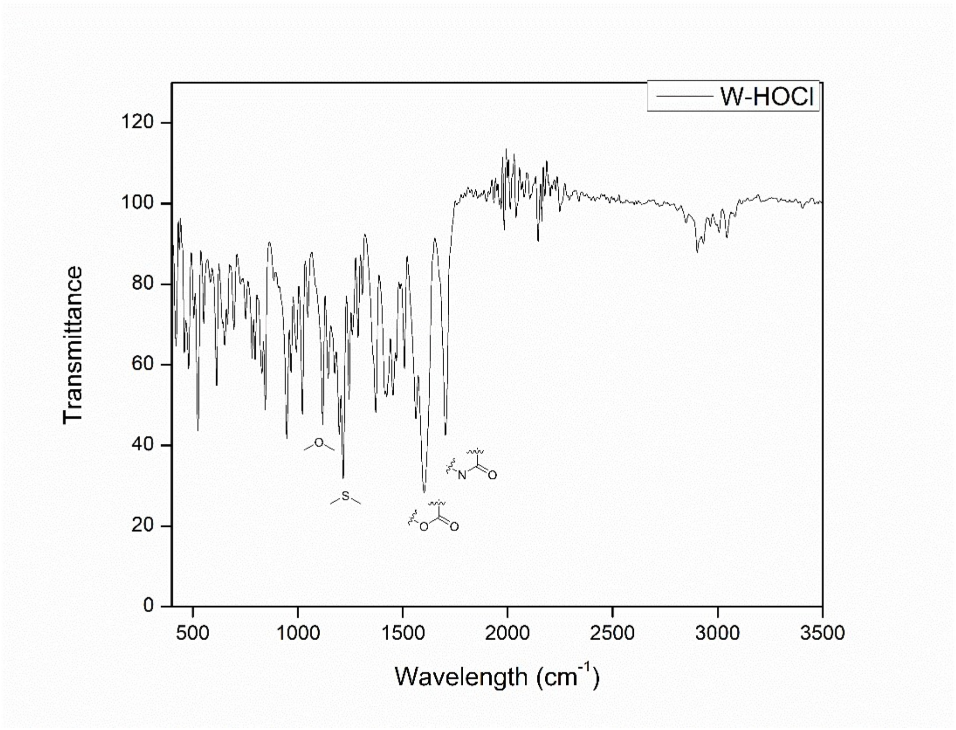

SI-3 IR of the probe W-HOCl

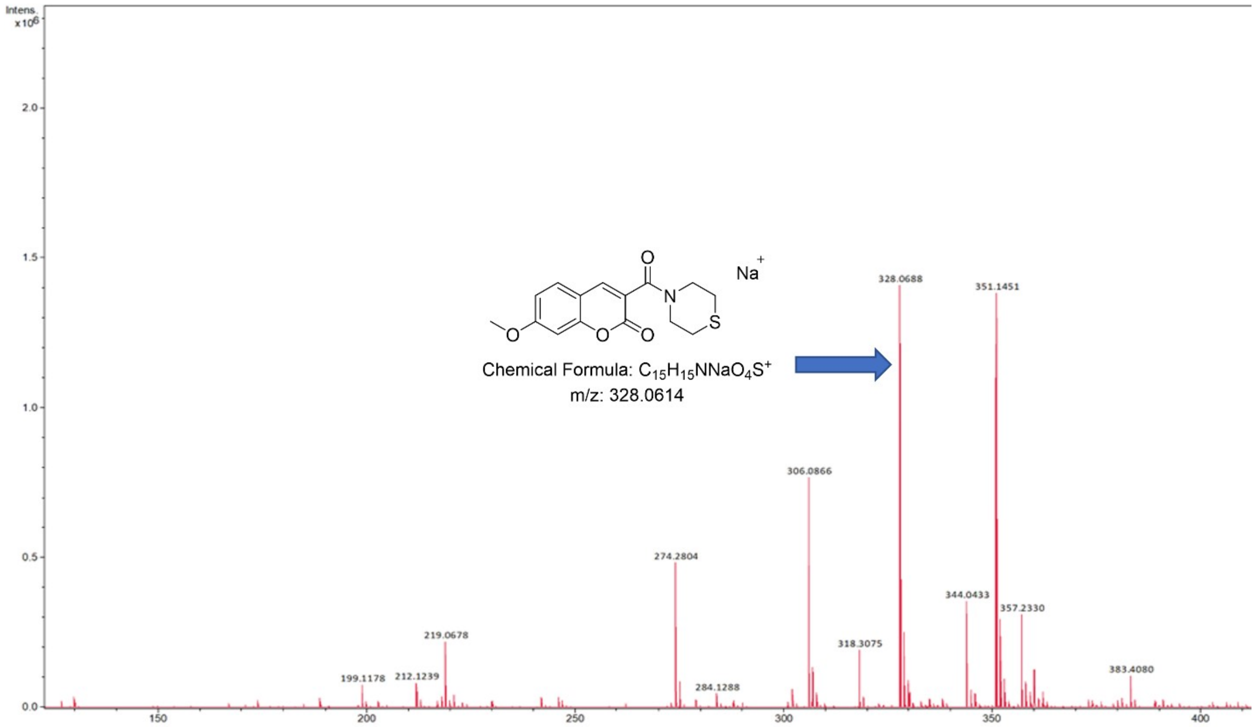

SI-4-HRMS of probe W-HOCl

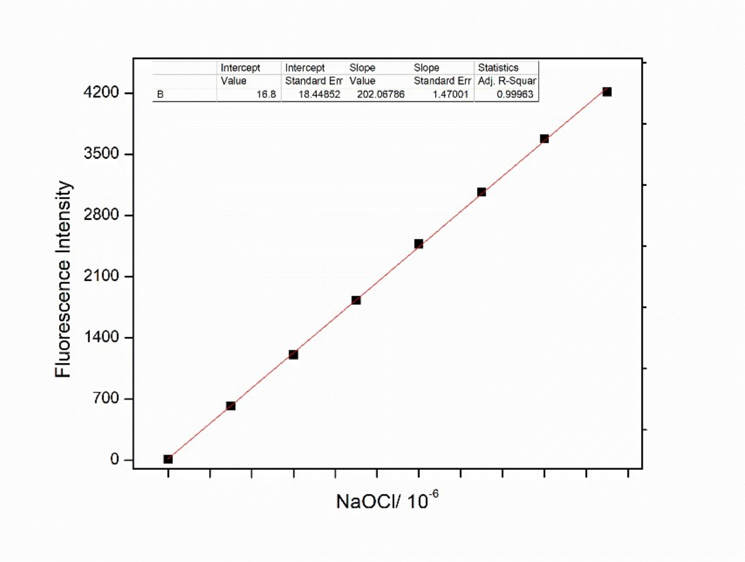

SI-5-linear plot of probe with NaOCl

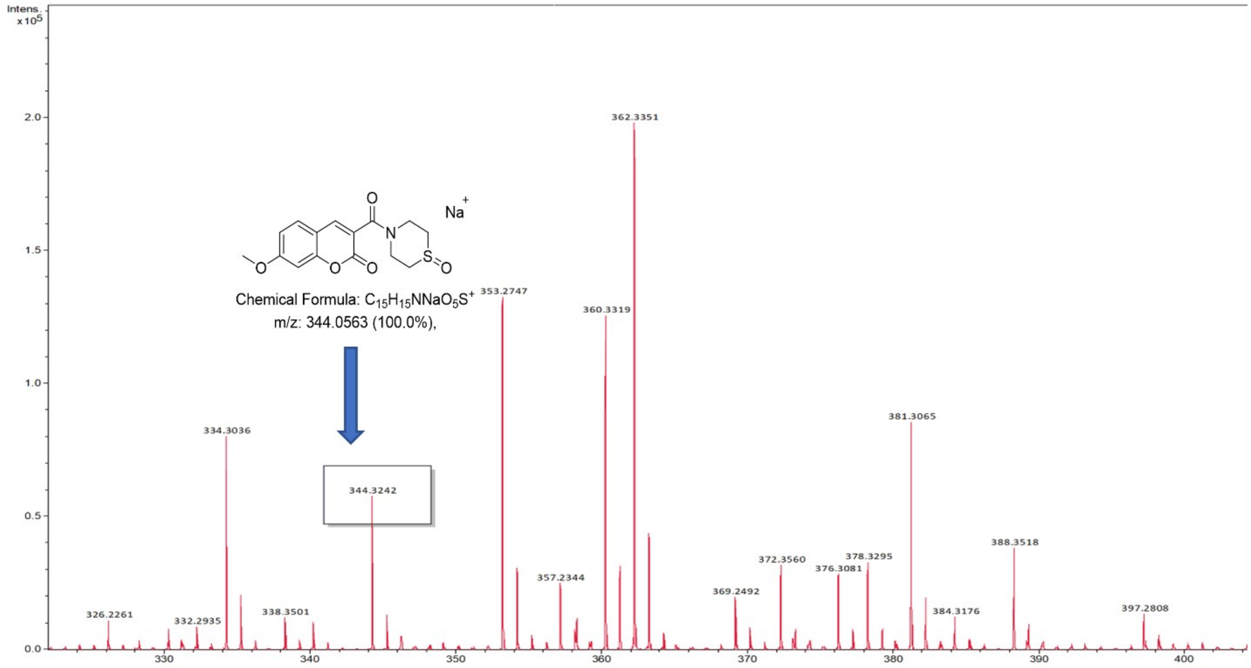

SI-6- HRMS of probe W-HOCl after adding HOCl

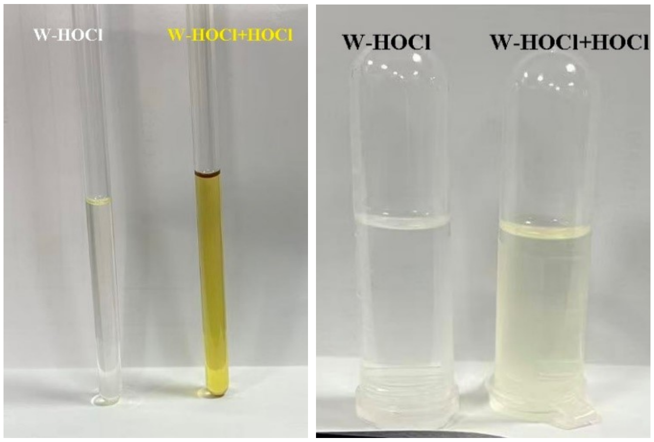

SI-7 Change in color after the addition of HOCl to probe solution and HNMR sample

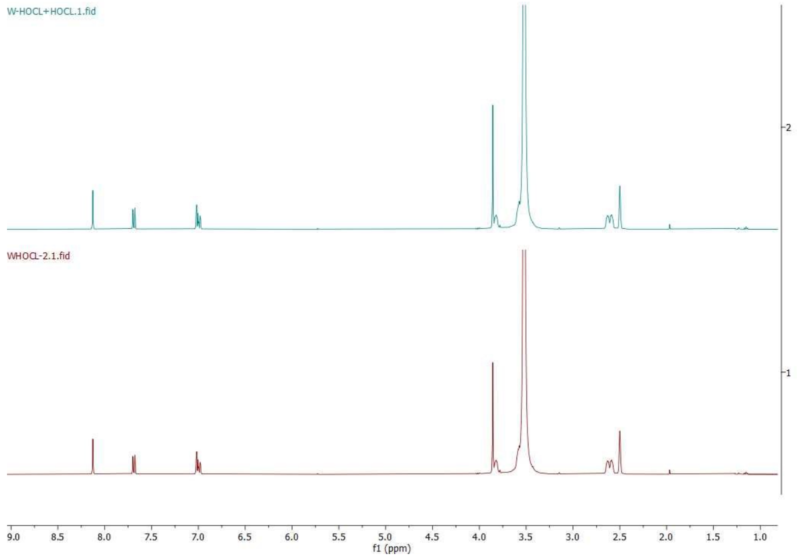

SI-8 Stability and proposed mechanism confirmation of probe through HNMR

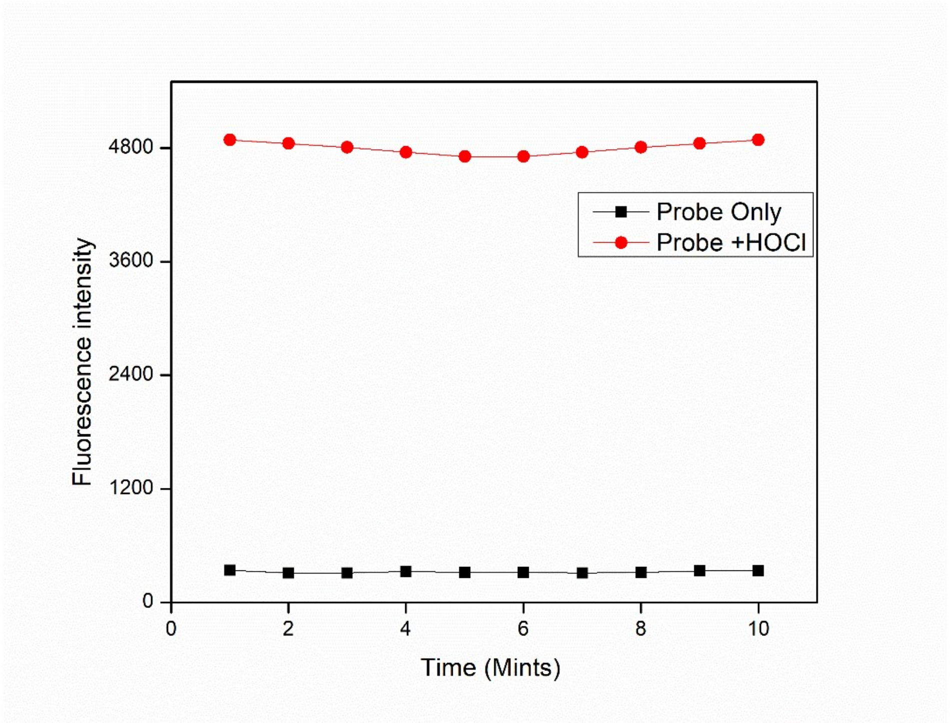

SI-9 Cycling test

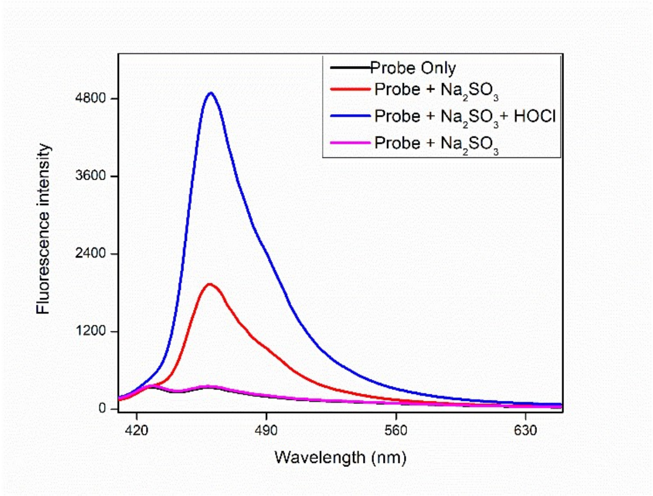

SI-10- Response of probe in excess of Na_2SO_3
